# A Systematic Review and Meta-Analysis on the Number of Adjuvant Temozolomide Cycles in Newly Diagnosed Glioblastoma

**DOI:** 10.3389/fonc.2021.779491

**Published:** 2021-11-23

**Authors:** Fahimeh Attarian, Farzad Taghizadeh-Hesary, Azar Fanipakdel, Seyed Alireza Javadinia, Pejman Porouhan, Babak PeyroShabany, Danial Fazilat-Panah

**Affiliations:** ^1^ Department of Public Health, School of Health, Torbat Heydariyeh University of Medical Sciences, Torbat Heydariyeh, Iran; ^2^ Department of Radiation Oncology, Shahid Beheshti University of Medical Sciences, Tehran, Iran; ^3^ Cancer Research Center, Mashhad University of Medical Sciences, Mashhad, Iran; ^4^ Vasei Clinical Research Development Unit, Sabzevar University of Medical Sciences, Sabzevar, Iran; ^5^ Department of Radiation Oncology, Sabzevar University of Medical Sciences, Sabzevar, Iran; ^6^ Department of Internal Medicine, Sabzevar University of Medical Sciences, Sabzevar, Iran; ^7^ Cancer Research Center, Babol University of Medical Sciences, Babol, Iran

**Keywords:** adjuvant, extended chemotherapy, glioblastoma, temozolomide, treatment duration, The Stupp protocol, high-grade gliomas

## Abstract

**Background:**

In newly diagnosed glioblastoma, radiation with concurrent and adjuvant (six cycles) temozolomide (TMZ) is the established standard of postsurgical care. However, the benefit of extending adjuvant TMZ therapy beyond six cycles has remained unknown.

**Methods:**

We searched PubMed, Web of Science, Scopus, and Embase up to October 1, 2021. The search keywords were “glioblastoma,” “adjuvant chemotherapy,” and their synonyms. The data of randomized clinical trials were extracted and included in this meta-analysis if they had reported patients’ median overall survival (OS) or median progression-free survival (PFS). The standard and extended chemotherapy regimens were considered as adjuvant TMZ up to six cycles and beyond six cycles (up to a total of 12 cycles), respectively. The median OS and median PFS were pooled and compared.

**Results:**

Four studies consisting of 882 patients (461 patients for the standard chemotherapy group and 421 patients for the extended chemotherapy group) were included in this meta-analysis. The extended TMZ regimen was associated with a nonsignificant improvement in PFS [12.0 months (95% CI 9.0 to 15.0) *vs*. 10.0 months (95% CI 7.0 to 12.0), *P* = 0.27] without corresponding improvement in OS [23.0 months (95% CI 19.0 to 27.0) and 24.0 months (95% CI 20.0 to 28.0), *P* = 0.73].

**Conclusions:**

In newly diagnosed glioblastoma, continuing adjuvant TMZ beyond six cycles did not shown an increase neither in PFS nor OS.

## 1 Introduction

Glioblastoma is the most common primary brain tumor of glial origin in adults. It is characterized by rapid progression, a high recurrence rate, and a dismal prognosis (1–3). Historically, the management of glioblastoma was maximal safe surgical resection (MSR) followed by radiotherapy. In the early 21^st^ century, a large randomized clinical trial (RCT) converted the standard of care to MSR, adjuvant chemoradiotherapy (CRT) [with concurrent temozolomide (TMZ), an oral alkylating agent], followed by TMZ for six cycles (4). Dismal prognosis of glioblastoma brought up the extended adjuvant TMZ (ext-TMZ) and changed the clinical practice to continue adjuvant TMZ up to 12 cycles or until tumor progression. Since then, numerous studies have attempted to compare the ext-TMZ and the standard Stupp protocol (std-TMZ). However, there is still no consensus on the duration of adjuvant TMZ (5).

In the English literature, there are several studies as case report (6), cohort study (7–18), clinical trial (19–24), and review article (5, 25, 26) assessing the potential benefits of ext-TMZ in patients with glioblastoma. In a pooled analysis of four RCTs (4, 21, 27, 28), the authors concluded that ext-TMZ did not improve the overall survival (OS) of patients with glioblastoma (26). This study did not include three recent RCTs (19, 20, 23). On the other hand, two other meta-analyses noted the improved OS and progression-free survival (PFS) with ext-TMZ (5, 25). These findings might be biased by their search strategy, as well as not including the recent RCTs. The present meta-analysis was therefore designed to compare the survival outcomes of std-TMZ and ext-TMZ in the first-line treatment of patients with glioblastoma.

## 2 Methods

### 2.1 Study Design and Types of Included Studies

This meta-analysis was conducted per the PRISMA (Preferred Reporting Items for Systematic Reviews and Meta-Analyses) guideline (29). It included RCTs comparing std-TMZ and ext-TMZ—as the first-line treatment in glioblastoma—in terms of median OS and median PFS.

### 2.2 Search Strategy

Two authors (S.A.J and F.A) independently searched the English literature for free-text and standard MeSH (Medical Subject Headings) terms in PubMed, Web of Science, Scopus, and Embase up to October 1, 2021. The search keywords were: high-grade glioma OR malignant glioma OR glioblastoma OR glioblastoma multiforme OR grade IV glioma OR grade IV astrocytoma OR GBM, adjuvant chemotherapy OR temozolomide OR temodar, AND extended OR long-term OR prolonged OR maintenance OR cycles OR months. Also, the two review authors handsearched the reference lists of the relevant articles to identify the possible missed RCTs. Thereafter, they downloaded all titles and abstracts retrieved by electronic searching to EndNote™ V.20.0, removed duplicates, and excluded the studies that did not meet the eligibility criteria, clearly (mentioned below). Eventually, they debated on the disagreements to improve the search results.

### 2.3 Study Screening

#### 2.3.1 Participants

Adult patients with glioblastoma who underwent surgery, radiotherapy (concurrent with TMZ), and adjuvant TMZ as the primary treatment.

#### 2.3.2 Inclusion Criteria

RCTs comparing std-TMZ and ext-TMZ, in which the median OS and/or median PFS were reported.

#### 2.3.3 Exclusion Criteria

Studies if (i) not following the standard treatment sequence of MSR, CRT (with TMZ), and adjuvant TMZ, (ii) submitted only as abstracts or proceedings from scientific meetings, (iii) lacking English full text or summaries, or (iv) including patients with recurrent glioblastoma were excluded. Eligible studies were assessed finally for quality of methodology (30, 31).

### 2.4 Data Extraction

The following data were extracted from the studies: (i) study information (the first author, year of publication, study country, sample size), (ii) patient baseline characteristics (age, sex ratio), (iii) intervention duration (std-TMZ, ext-TMZ), and (iv) treatment outcomes (median OS, median PFS). Only data from the first-line therapy of both groups were extracted.

The standard chemotherapy regimen (std-TMZ) was defined as ≤ 6 cycles of TMZ following MSR and adjuvant CRT. The extended chemotherapy regimen (ext-TMZ) was defined as > 6 cycles of TMZ (up to 12 cycles) following MSR and adjuvant CRT. The median OS and PFS were extracted directly from the text or the Kaplan-Meier survival curves.

### 2.5 Quality Assessment

Two investigators (S.A.J. and F.A) assessed the methodological quality and risk of bias of the included studies. All four included studies were assessed using Cochrane’s Risk of bias tool (31). They resolved differences by discussion or appeal to a third review author (F.T) and presented results in a “Risk of bias” table. The risk of bias summary consists of 5 questions (also known as the Oxford quality scoring system), ranging from 0 to 5. Studies with a quality score less than 3 were regarded as poor quality and excluded from the study (**Table 1**).

**Table 1:** Methodological quality summary for the included studies.

Risk of bias	Balana, 2020	Bhandari, 2017	Blumenthal, 2017	Refae, 2015
Random sequence generation (selection bias)	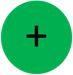	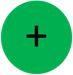	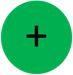	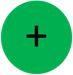
Allocation concealment (selection bias)	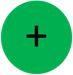	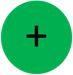	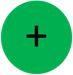	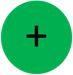
Blinding (performance bias and detection bias)	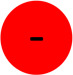	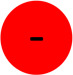	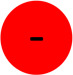	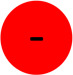
Incomplete outcome data (attribution bias)	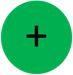	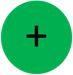	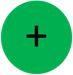	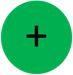
Selective reporting (Reporting bias)	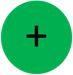	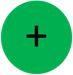	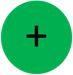	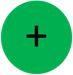

+ means that the corresponding article (in column) consider the criteria (the row) or not (-).

### 2.6 Statistical Analysis

The main objective of this meta-analysis was to compare the median PFS and OS for std-TMZ versus ext-TMZ as the first-line treatment of patients with glioblastoma. The individual patient data (IPD) is essential in the standard approach to pooled survival estimates (26). However, IPD was unavailable in this meta-analysis, and we used the median PFS and OS (weighted by the inverse of variance) to estimate the pooled median and 95% confidence interval (95% CI) of PFS and OS in each group of RCTs. The statistical heterogeneity between studies was evaluated using Cochran’s Q test and quantified by I^2^ statistics (high heterogeneity was defined as I² > 20% or P-value < 0.1). We applied Stata V.14.0 (Stata Corp, College Station, TX, USA) for the quantitative synthesis. The statistical significance level was set to 0.05.

## 3 Results

**Figure 1:**
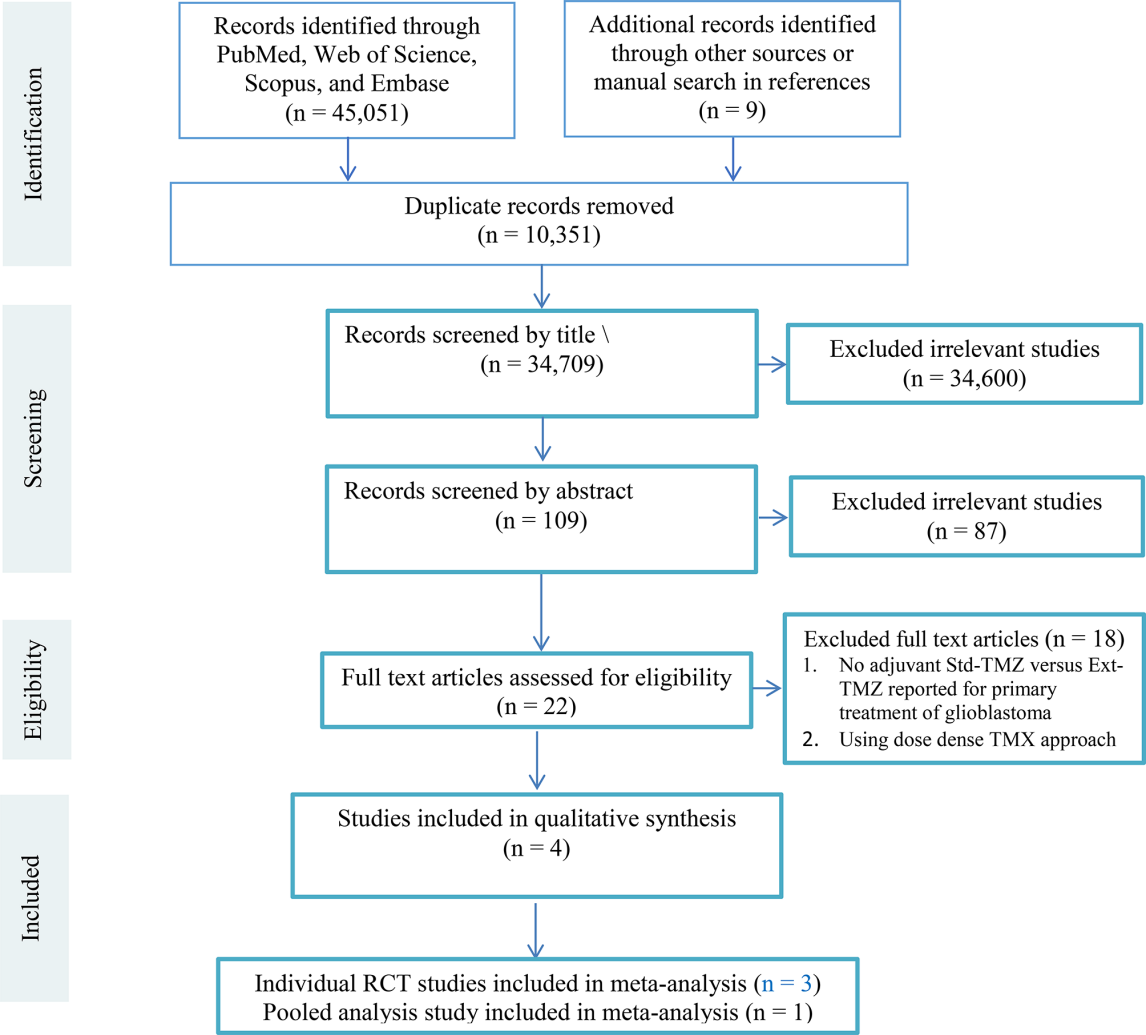
The PRISMA flow diagram. Three out of four eligible studies were randomized comparisons of adjuvant std-TMZ versus ext-TMZ for primary treatment of glioblastoma (19, 20, 23).The remaining study was a pooled analysis of four RCTs (26). The main characteristics of the eligible studies are shown in **Table 2**.

Our databases searching identified 45,060 potentially relevant studies. After deleting duplicates (10,351 records), 22 articles were included in the evaluation through screening titles, abstracts, and full texts. Then, we excluded eighteen studies (4–10, 12–17, 21, 22, 24, 32) on eligibility criteria. **Figure 1** details the PRISMA flow diagram.

**Table 2:** Main characteristics of four studies included in the current meta-analysis.

ID	First Author, year	Country	Study design	Study population	Age distribution (years old)	Sex ratio (M/F)	No. of TMZ cycles (n)	
							≤6	>6
1	Balana, 2020	Spain	RCT	159	≥ 18	83/76	79	80
2	Bhandari, 2017	India	RCT	40	18-65	24/16	20	20
3	Blumenthal, 2017	International	RCT	624	N/A	354/270	333	291
4	Refae, 2015	Egypt	RCT	59	19 – 72	47/12	29	30
Total				882	≥ 18	508/374	461	421

RCT, randomized clinical trial; TMZ, temozolomide; NA, not available.

**Figure 2:**
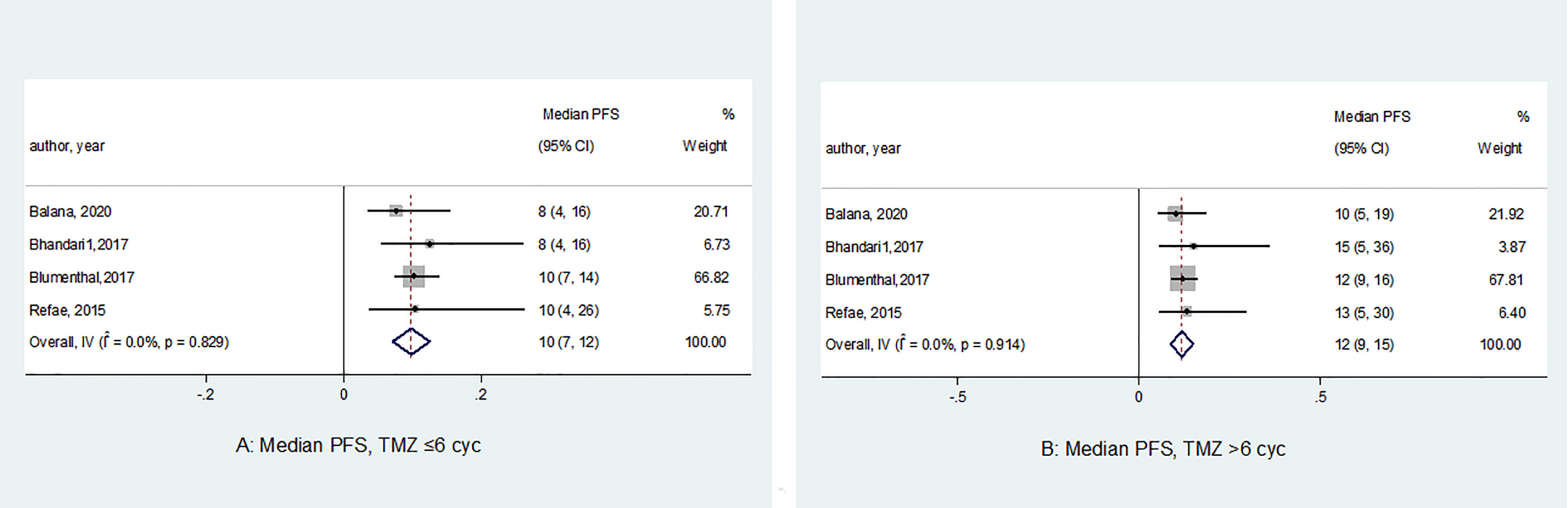
Forest plots of the median progression-free survival (PFS) according to the number of adjuvant temozolomide cycles. The horizontal line of the diamond summary represents the average 95% CI. The statistical heterogeneity between studies was assessed using the I^2^ test, which revealed a homogeneity in the results (PFS in the std-TMZ, I^2^ = 0.0%, *P* = 0.82; and PFS in the exd-TMZ, I^2^ = 0.0%, *P* = 0.91).

A total of 882 glioblastoma patients were included in the four studies. Of these, 461 patients were treated by std-TMZ regimen, and 421 patients received ext-TMZ regimen. The median PFS of patients with glioblastoma who were treated by the standard or extended chemotherapy regimens is shown in **Figure 2**. The overall median PFS was 10.0 months (95% CI 7.0 to 12.0) in the std-TMZ group (TMZ ≤ 6 cycles). The studies had homogeneity in median PFS in this group (*P* = 0.82). Likewise, in the ext-TMZ group, all studies had homogeneity in the median PFS 12.0 months (95% CI 9.0 to15.0 months) (*P* = 0.91). The least record of median PFS (10.0 months 95% CI 5.0 to 19.0) in arms with ext-TMZ (TMZ > 6 cycles) was equal to the upper record of median PFS in the std-TMZ group (10.0 months 95% CI 4.0 to 26.0). Comparison between the two groups showed that ext-TMZ was associated with an improved PFS (12.0 months, 95% CI 9.0 to 15.0 *vs*. 10.0 months, 95% CI 7.0 to 12.0), although this improvement was not statistically significant (*P* = 0.27) (**Figure 2**).

**Figure 3:**
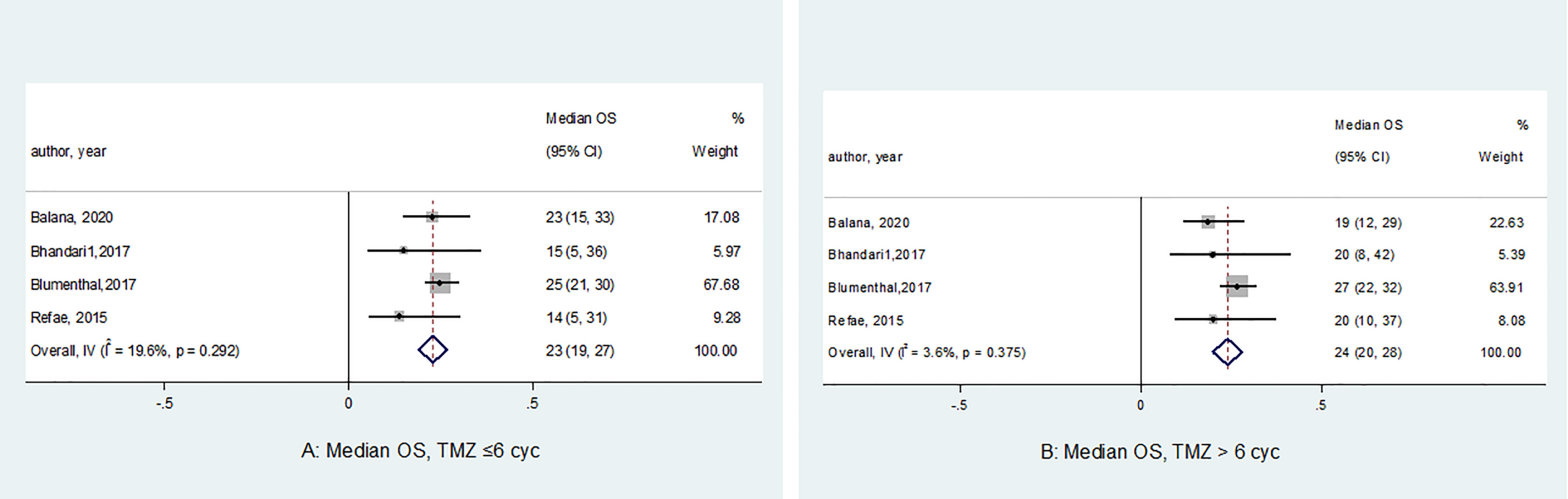
Forest plots of the median overall survival (OS) according to the number of cycles of adjuvant temozolomide (*P* = 0.99). The horizontal line of the diamond summary represents the average 95% CI. The statistical heterogeneity between studies was assessed using the I^2^ test, which revealed homogeneity in the results (OS in the std-TMZ, I^2^ = 19.6%, *P* = 0.29;and OS in the ext-TMZ, I^2^ = 3.6%, *P* = 0.37).

The median OS of the analyzed studies ranged from 14.0 to 25.0 months for the std-TMZ group (n = 461) versus 19.0 to 27.0 months for the ext-TMZ group (n = 421) (**Figure 3**). Three out of four studies reported superior median OS in the ext-TMZ group; however, Balana et al. found the contrast results (median OS: 19.0 months in ext-TMZ *vs*. 23.0 months in std-TMZ). The pooled estimated median OS of patients in the std-TMZ and ext-TMZ were statistically consistent [23.0 months (95% CI 19.0 to 27.0) and 24.0 months (95% CI 20.0 to 28.0), respectively, *P* = 0.73].

## 4 Discussion

This meta-analysis assessed the survival benefit of adjuvant ext-TMZ (7-12 cycles) against the standard 6-cycle regimen for patients with newly diagnosed glioblastoma. The literature search yielded four studies (three RCTs and one pooled analysis) that met our eligibility criteria, including 461 patients in the std-TMZ group and 421 patients in the ext-TMZ group. The quantitative analysis showed trend, although nonsignificant, towards improved PFS with the ext-TMZ regimen [12.0 months (95% CI 9.0 to 15.0) *vs.* 10.0 months (95% CI 7.0 to 12.0), *P* > 0.05]. However, the OS of patients who were treated by the ext-TMZ and the std-TMZ remained almost identical [23.0 months (95% CI 19.0 to 27.0) *vs*. 24.0 months (95% CI 20.0 to 28.0), *P* > 0.05, respectively]. These findings are inconsistent with the previous meta-analyses by Alimohammadi et al. and Xu et al., stating that extended adjuvant TMZ improves both PFS and OS in patients with newly diagnosed GBM (5, 25). However, these findings might be affected by the included retrospective records, which constituted 28.9% and all of the analyzed cases in the Alimohammadi et al.’s and Xu et al.’s studies, respectively. In our analysis, we excluded all retrospective studies to enhance the power of the results.

In summary, the current meta-analysis did not demonstrate the survival benefit of prolonged adjuvant TMZ in newly diagnosed glioblastoma. This might be explained by adaptive resistance. To better understand this issue, we need to recognize the mechanism of action of TMZ. As an alkylating agent, TMZ acts as a prodrug and induces cell cycle arrest at G_2_/M through methylation of DNA or RNA. The methylated sites can remain mutated by DNA mismatch repair (MMR) proteins, dealkylated by the action of O^6^-methylguanine methyltransferase (MGMT), or be removed by the base excision repair (BER) enzymes [such as alkylpurine-DNA-N-glycosylase (APNG)]. Cells are TMZ sensitive when MMR is overexpressed and active. On the other hand, MGMT or BER proteins overexpression increases the resistance of glioblastoma cells to TMZ. *In vitro* studies have delineated several mechanisms of adaptive resistance to TMZ in glioblastoma cell lines. For example, increased MGMT protein expression (33), decreased Tumor Necrosis Factor-Alpha-Induced Protein 3 (TNFAIP3) expression (34), upregulation of Signal Transducer and Activator of Transcription 3 (STAT3) (35), loss of MSH6 MMR gene (36), or upregulation of NTL1 (a BER enzyme) (37). Therefore, prolonged adjuvant TMZ can promote the development of tumor-resistant clones with more aggressive features. This issue can contribute to a dismal prognosis in salvage therapy of tumor recurrence. A multicenter, phase II trial—evaluating the efficacy of continuous dose-intense TMZ for recurrent glioblastoma—concluded that patients who had received adjuvant std-TMZ got more benefit from therapy in comparison with ext-TMZ group (38).

In addition to the idiosyncratic adverse effects of TMZ (such as aplastic anemia, cholestatic hepatitis, and myelosuppression), clinicians must consider the numerous intrinsic adverse effects of TMZ that might affect the quality and quantity of life of the patients with glioblastoma. In this regard, different retrospective studies have reported different rates of toxicities, as follows: lymphopenia (30-50%), nausea (28-44.3%), vomiting (20-37%), fatigue (10-33%), anorexia (14%), thrombocytopenia (12-13.7%), anemia (1-11%), neutropenia (6.3-7%), leukopenia (1.3-7%), myelodysplasia, or leukemia. The diverse rates of TMZ adverse effects might be due to different distribution of basic characteristics (vomiting and thrombocytopenia are more common in females), stage of treatment (hematological toxicities are more common in the concurrent chemoradiation phase), and the numbers of adjuvant TMZ cycles. In the context of lymphopenia, TMZ can increase the risk of opportunistic infections (such as pneumocystis jiroveci pneumonia, herpes zoster, candida) through selective CD4^+^ T-cell depletion (39, 40). The intrinsic adverse effect of TMZ is another evidences to avoid prolonged adjuvant TMZ. In an RCT, patients in the ext-TMZ arm experienced more grade ≥ 3 hematological toxicities (5% *vs*. none), vomiting (15% *vs*. 10%), and insomnia (10% *vs*. 5%) in comparison to the std-TMZ regimen. However, the rates of fatigue and headache were more prevalent in the std-TMZ arm (50% *vs*. 45% and 15% *vs*. 10%, respectively) (20).

Our study harbors several limitations. Lack of access to IPD and not reporting the hazard ratio in most of the studies are among the main ones. Besides, the current evidence on the role of MGMT methylation in the value of prolonged TMZ therapy beyond six months is either not addressed properly in the clinical trials (20, 23), or is assessed retrospectively (26). Therefore, prospective data on the predictive value of MGMT methylation is lacking, and our analysis cannot provide any comment in this regard. Among the included studies, only Balana et al. evaluated the role of MGMT on the survival of patients with glioblastoma receiving first-line adjuvant TMZ. By multivariate analysis, they showed that MGMT methylation was an independent factor for longer PFS and OS. However, this finding was not translated into the survival benefit of extended TMZ in patients with MGMT methylation (19).

In conclusion, prolonged adjuvant TMZ (beyond six cycles) did not provide OS and PFS benefits in patients with newly diagnosed glioblastoma. Considering this finding, along with the adverse effects of TMZ, the economic burden and psychosocial impacts of prolonged treatment can underscore the rationality of the current practice, which is 6 cycles of adjuvant TMZ. Further studies are needed to determine the predictive value of MGMT status on the long-term TMZ maintenance therapy. Moreover, the role of surgically validated results of dynamic imaging such as O-(2-[^18^F] fluoroethyl-)-L-tyrosine positron emission tomography (^18^F-FET PET) on the extending of TMZ should be assessed in future studies.

## Data Availability Statement

The original contributions presented in the study are included in the article/supplementary material. Further inquiries can be directed to the corresponding author.

## Author Contributions

SJ, FT-H, and FA designed the meta-analysis, screened the studies and wrote the manuscript. FA analyzed data. AF, PP, and BP helped designed the study. All authors reviewed the manuscript finally. DF-P and AF wrote the initial draft. SJ, FA, PP, and BP approved the final submission of the manuscript.

## Conflict of Interest

The authors declare that the research was conducted in the absence of any commercial or financial relationships that could be construed as a potential conflict of interest.

## Publisher’s Note

All claims expressed in this article are solely those of the authors and do not necessarily represent those of their affiliated organizations, or those of the publisher, the editors and the reviewers. Any product that may be evaluated in this article, or claim that may be made by its manufacturer, is not guaranteed or endorsed by the publisher.
